# Metasurface Broadband Solar Absorber

**DOI:** 10.1038/srep20347

**Published:** 2016-02-01

**Authors:** Abul K. Azad, Wilton J. M. Kort-Kamp, Milan Sykora, Nina R. Weisse-Bernstein, Ting S. Luk, Antoinette J. Taylor, Diego A. R. Dalvit, Hou-Tong Chen

**Affiliations:** 1 Center for Integrated Nanotechnologies, Los Alamos National Laboratory, MS K771, Los Alamos, New Mexico 87545, USA; 2 Theoretical Division, Los Alamos National Laboratory, MS B213, Los Alamos, New Mexico 87545, USA; 3 Center for Nonlinear Studies, Los Alamos National Laboratory, Los Alamos, New Mexico 87545, USA; 4 Chemistry Division, Los Alamos National Laboratory, MS K558, Los Alamos, New Mexico 87545, USA; 5 Intelligence and Space Research Division, Los Alamos National Laboratory, MS B244, Los Alamos, New Mexico 87545, USA; 6 Center for Integrated Nanotechnologies, Sandia National Laboratories, Albuquerque, New Mexico 87123, USA

## Abstract

We demonstrate a broadband, polarization independent, wide-angle absorber based on a metallic metasurface architecture, which accomplishes greater than 90% absorptance in the visible and near-infrared range of the solar spectrum, and exhibits low absorptivity (emissivity) at mid- and far-infrared wavelengths. The complex unit cell of the metasurface solar absorber consists of eight pairs of gold nano-resonators that are separated from a gold ground plane by a thin silicon dioxide spacer. Our experimental measurements reveal high-performance absorption over a wide range of incidence angles for both s- and p-polarizations. We also investigate numerically the frequency-dependent field and current distributions to elucidate how the absorption occurs within the metasurface structure.

Metamaterials have allowed the demonstration of many exotic electromagnetic phenomena and inspired some interesting potential applications^1^. While bulk metamaterials pose severe fabrication challenges particularly in the optical regime^2^, planar metamaterial architectures – metasurfaces – offer alternative avenues to accomplish desirable functionalities, including the manipulation of wavefront^3^, polarization conversion^4^, and absorption/emission engineering^5^. Metasurface perfect absorbers with thickness much smaller than the operational wavelength are attractive in many applications such as sensing^6^, compressive imaging^7^, and thermal management^8^,^9^.

Broadband absorbers covering the entire solar spectrum are also of great interest in solar energy harvesting^10^. There have been some demonstrations of material structures as high-performance solar absorbers, for instance, using dense nanorods and nanotube films^11^,^12^, multilayer planar photonic structures^8^,^13^,^14^, and photonic crystals^15^. Metasurfaces consisting of complex multi-resonator unit-cells have emerged as a powerful and flexible platform to realize multiband and broadband perfect absorption in the microwave^16^, terahertz^17^, infrared^18^,^19^,^20^, and visible^21^,^22^,^23^ regimes. Extending the absorption bandwidh of a solar absorber, covering entire visible and near-IR spectrum with a controllable cutoff wavelength for minimizing mid- and far-IR emmisivity, is highly desirable.

Inspired by these earlier works, here we demonstrate the design, fabrication and characterization of a broadband wide-angle metasurface absorber exhibiting a solar weighted absorptance of 88% in the wavelength range 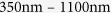, and less than 2% absorptance (emissivity according to Kirchhoff’s law) at wavelengths larger than 1500 nm. The relatively simple design of the absorber allows scale up to large area fabrication using conventional nano-imprint lithography.

## Results and Discussion

**Figure 1:**
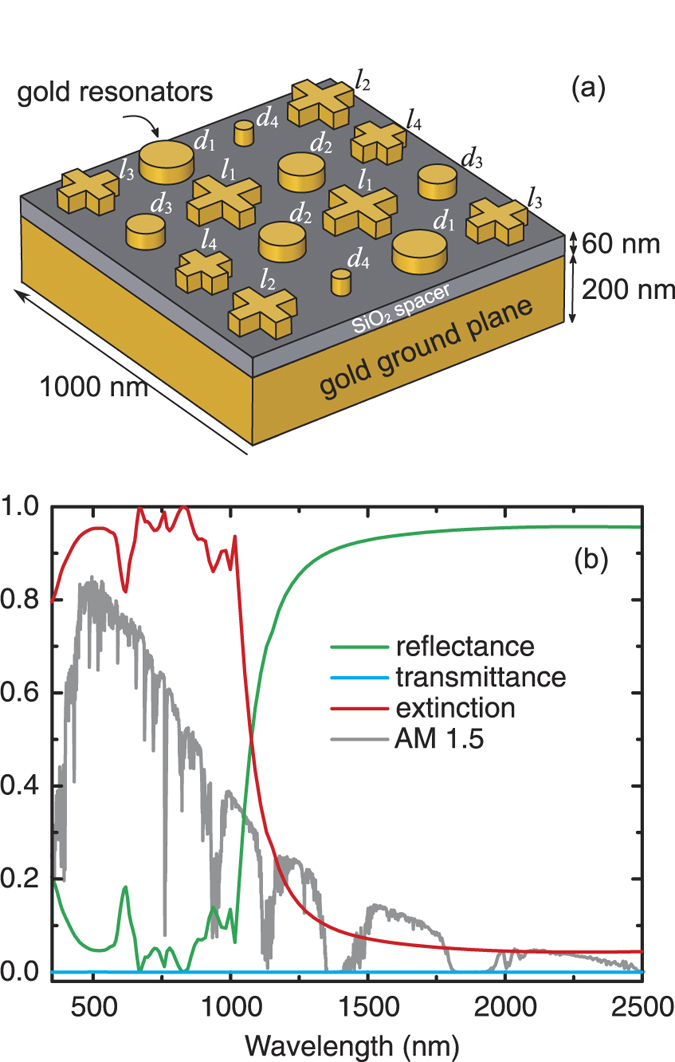
(**a**) Schematic representation of the super-cell of the broadband metasurface absorber consisting of 16 resonant elements forming a square array. The lengths of the crosses are *l*_*1*_ = 200 nm, *l*_*2*_ = 180 nm, *l*_*3*_ = 160 nm, and *l*_*4*_ = 140 nm, and all crosses have identical arm widths *w* = 50 nm. The diameters of the circles are *d*_*1*_ = 140 nm, *d*_*2*_ = 120 nm, *d*_*3*_ = 100 nm, and *d*_*4*_ = 50 nm. (**b**) Simulated reflectance, transmittance, and extinction spectra at normal incidence. The gray curve shows the AM1.5 solar spectrum, normalized to fit the scale of the plot.

The schematic diagram of our metasurface absorber is illustrated in Fig.1(a), which is based on a metal-dielectric-metal architecture. It consists of an array of 50 nm thick gold nano-resonators and a 200 nm thick gold ground plane separated by a 60 nm thick silicon dioxide spacer. A super-cell containing sixteen resonators of different sizes and shapes was employed to enable broadband absorption, and they all have four-fold symmetry to provide a polarization independent response. The design was first validated through numerical simulations using commercially available full-wave electromagnetic solvers (CST Microwave Studio and COMSOL Multiphysics). It is worth noting that high absorption can also be achieved by using super-cells consisting of single-shaped resonators (e.g. crosses or squares) of different sizes, as was done in ref. 23 for the visible spectrum. However, we opted to use a combination of shapes (crosses and circles) in order to have more degrees of design-freedom in tailoring the absorption spectrum, e.g., the sharpness of the cut-off wavelength in the near IR spectral region. The dimensions of the resonators, their spatial distribution, and the thickness of the spacer were tuned to optimize the absorption performance within the desired spectral window. Additionally, the performance may be further improved by designing more sophisticated unit cells using optimization methods, e.g. genetic algorithms^19^.

Figure 1(b) shows the simulated reflectance *R*, transmittance *T*, and extinction 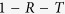 under normal incidence. When the possible scattering to directions other than specular reflection directions is negligible, this extinction is equivalent to absorptance. This is confirmed in our experiments where the scattering is beyond the detection limit of our instrument. Due to the thick gold ground plane, the transmittance through the structure is essentially zero. The simulation reveals that our structure exhibits over 90% absorptance approximately in the 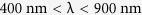 spectral range, and a near-zero absorption at longer wavelengths with a cutoff wavelength 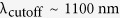 .

**Figure 2:**
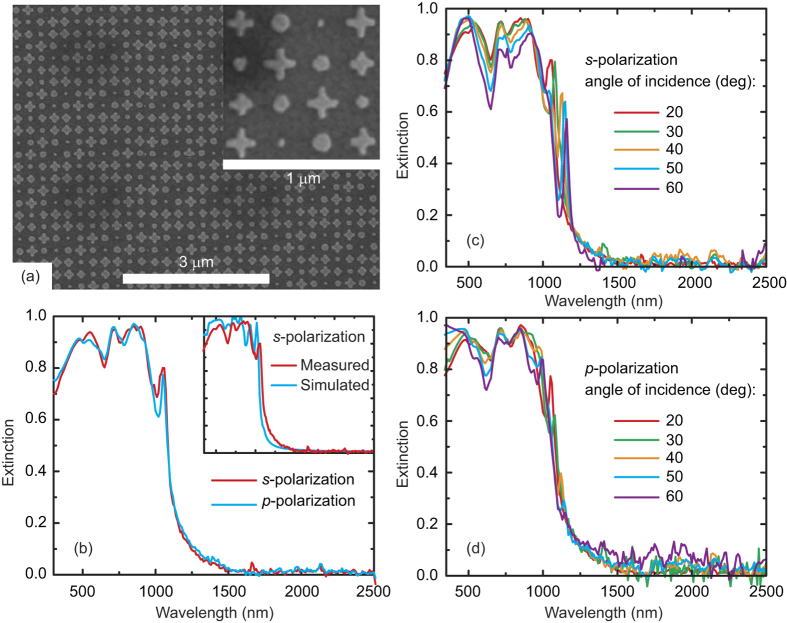
(**a**) SEM image of a portion of the fabricated absorber. The inset shows an expanded view of the super-cell. (**b**) Experimentally measured extinction for *s*- and *p*- polarizations at 20° angle of incidence, and at various angles of incidence for *s-* (**c**) and *p-*polarized (**d**) light. Inset to (**b**) is a comparison between experiments and simulations both at 20° angle of incidence for *s*-polarized incident light.

The metasurface absorber was fabricated on a silicon substrate that provides the necessary mechanical support. A scanning electron microscopy (SEM) image of the sample is shown in Fig. 2(a), where the inset depicts an expanded view of the super-cell. The image shows slight deviations from the original design due to the fabrication tolerance, as revealed by, e.g., the rounded corners of the cross resonators. The active area of the fabricated metasurface absorber is 450 μm × 450 μm. The sample was characterized at wavelengths between 350 nm and 2.5 μm using a J. A. Woollam variable angle spectroscopic ellipsometer (VASE).

The measured absorptance at 20° angle of incidence is shown in Fig. 2(b), for both *s-* and *p*-polarizations, confirming the polarization-independent high absorption over almost the entire solar spectrum. In the wavelength range 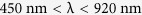 our metasurface absorber accomplishes absorptance higher than 90%. The broadband absorption has a sharp edge near 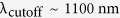, and the solar weighted absorptance is 88% in the wavelength range 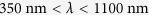. The absorptance becomes less than 10% when 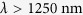 , and at wavelengths above 1500 nm, the measured absorptance is negligible (<2%), while the simulations at the same incidence angle exhibit higher values (~4%) when using tabulated dielectric properties of gold^24^. In order to understand the origin of this discrepancy, we experimentally measured the dielectric properties of our gold film using ellipsometry and employed them in additional simulations. The results for the case of *s*-polarized incident light are shown in the inset to Fig. 2(b), revealing an improved agreement between measurements of our fabricated sample and simulations of the designed structure despite the fabrication tolerance; for *p*-polarization the agreement is similar. Taking into account in the simulations the rounded corners of the cross resonators and the slight variations of the diameters of the circles does not significantly change this good agreement, which also confirms the robustness of our metasurface absorber. The broadband and high absorption performance is maintained even when the angle of incidence increases up to 60°, as shown in the measured absorptance plotted in Fig. 2(c,d) for *s*- and *p*-polarized light, respectively. At these angles of incidence, we also confirmed numerically that the absorption performance remains practically unchanged while changing the sample azimuthal angle.

**Figure 3:**
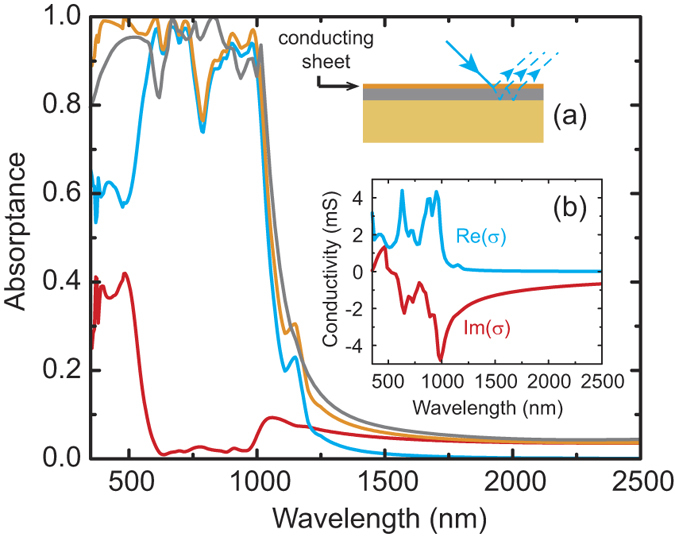
Absorptance spectra at normal incidence calculated using full-wave simulations (gray) and homogenization approximation (orange). Also shown are the contributions to the absorptance due to the resonators (cyan) calculated using Eq. (1) and ground plane (red) based on Eq. (2) (see Methods). Inset (**a**) is a schematic of the multiple reflection process that takes place within the absorber, and inset (**b**) shows the calculated effective conductivity of the sheet containing the resonator array.

Our metasurface achieves broadband absorption through a combination of resonators with different geometries in a super-cell, which provides a complex dispersion that enables Fabry-Pérot destructive interference in reflection at multiple frequencies, causing light trapping and high absorption^25^. For appropriately designed structures with these frequencies sufficiently close together, broadband absorption becomes possible, particularly in the optical wavelength range where metals are lossy. On the other hand, it is unavoidable that at some wavelengths the conditions of complete destructive interference are less satisfied due to the limited number of resonance modes, resulting in absorption dips as can be seen in Figs 2 and 3. Owing to the complex profile of the metasurface, it is difficult to derive an exact analytical expression for its reflectance, transmittance, and absorptance. Instead, we use a homogenization approach in which we determine an effective conductivity of the planar resonator array by enforcing that the numerically calculated reflection and transmission coefficients of the resonator array/spacer system correspond to those of the spacer slab coated with a 2D homogeneous sheet with conductivity *σ*; the resulting conductivity obtained by this procedure is shown as the inset (b) to Fig. 3. Then our metasurface absorber is equivalent to the multilayered system as shown in the inset (a) to Fig. 3, where for simplicity we have assumed a semi-infinite ground plane due to the small penetration depth of light into the gold ground plane. We compare in Fig. 3 the absorptance obtained through this homogenization technique with the exact full-wave numerical results (using tabulated dielectric properties of gold^24^), which exhibits an excellent agreement. The slight mismatch is due to the fact that the approximation neglects the interaction of the resonators with the ground plane^26^. Using this approach one can easily compute the absorption within the resonator array and the ground plane (we neglect any absorption within the spacer because silicon dioxide is almost lossless at the wavelengths of interest). We plot these absorptance spectra in Fig. 3, which reveals that for wavelengths less than 500 nm both the resonator array and the ground plane contribute to the total absorbed power, while the contribution of the resonator array to the absorption mechanism becomes more dominant in the range 500–1000 nm. In this range the ground plane absorbs negligible light and works as a perfect reflecting mirror. As a consequence, the high absorptance (>90%) of the metasurface at these wavelengths relies on the superposition of multipolar resonances excited within the resonators. At longer wavelengths the light absorption is dominated by the contribution from the ground plane. Finally, we should emphasize that although the homogenization approach enables an analytical understanding of the absorption mechanism in the metasurface absorber, it requires the numerically computed reflection and transmission of the resonator array.

**Figure 4:**
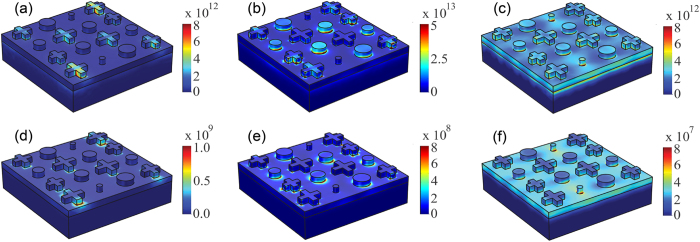
Simulated moduli of current (top) and electric field (bottom) on the surface of the broadband metasurface absorber for impinging wavelengths of 1000 nm (**a,d**), 705 nm (**b,e**), and 350 nm (**c,f**). Units of current and electric field are A/m^2^ and V/m, respectively.

To better understand the broadband nature and how energy is dissipated in our metasurface absorber, we investigated the surface current and the electric field spatial distributions. At wavelengths λ > 1100 nm, the incident light does not excite any resonators and the whole structure acts as a highly reflecting mirror; when reaching the cutoff wavelength 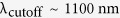 the resonators start to be excited. For instance, at λ~1000 nm only the larger cross resonators are excited – the distributions of the current ***J***, the electric field ***E***, and the corresponding absorption 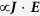 are mainly concentrated within them, as shown in Fig. 4(a,d). Medium-sized resonators are excited and dominate the absorption process at intermediate wavelengths, as shown in Fig. 4(b,e) for λ = 705 nm. At shorter wavelengths all resonators contribute to the overall absorptance, and the smaller ones provide the largest contribution, as shown in Fig. 4(c,f) for λ = 350 nm. Also note that at these wavelengths the light field penetrates more into the gold ground plane, and the induced currents result in a non-negligible contribution of the ground plane to the overall absorptance.

## Conclusions

In conclusion, we have demonstrated a metallic metasurface absorber that enables very high absorption over the energy-rich portion of the solar spectrum and low thermal radiation at mid- and far-infrared wavelengths. This is accomplished through a deliberately designed metasurface super-cell consisting of multiple resonators that are excited over the wavelength range of interest. Our experiments are in excellent agreement with full-wave simulations.

## Methods

### Numerical simulations

The full wave numerical simulations were carried out using periodic boundary conditions and frequency dependent tabulated dielectric properties of gold^24^ and silicon dioxide^27^. We obtained the S parameters (S_21_ for transmission and S_11_ for reflection) and the extinction was derived using, 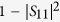 as the transmission (S_21_) is zero due to the thick gold ground plane.

### Sample fabrication and characterization

The absorber was fabricated using standard electron beam lithography. A 200 nm thick gold film was first deposited on silicon wafer using electron beam evaporation, followed by chemical vapor deposition of a 60 nm thick silicon dioxide film. The array of 50 nm thick gold nano-resonators was then created using electron beam lithography, metal deposition, and a lift-off process. The fabricated sample was measured using an optical spectrometer that allowed wavelength-dependent high-accuracy reflection and transmission measurements, with the optical beam focused down to ~200 μm in diameter and for angle of incidence limited to 20°–70° due to mechanical constraints of the instrument. Sample reflection spectra were normalized to reference spectra from a gold mirror, from which we derived the sample extinction. Non-specular reflection is below the detection limit of our system; therefore we assume the extinction equals the absorptance.

### Theoretical analysis

The absorbed power by the 2D sheet (resonator array) is given by 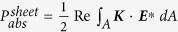 , where ***K = **σ**E*** is the surface current density, ***E*** is the electric field at the sheet, and *A* is the area. The absorptance of the sheet is then given by:


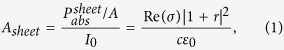


where *I*_*0*_ is the incident intensity, *r* is the reflection coefficient of the multilayered system, *c* is the speed of light in vacuum, and ε_0_ is the vacuum permittivity. The power dissipated within the ground plane is given by 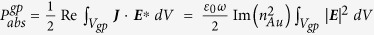 , where 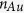 is the complex refractive index of gold, and the integration extends over the entire volume of the ground plane. However, since the ground plane acts as an almost perfect mirror, we can approximate the field by its value at the interface spacer-ground plane and restrict the integral to the volume 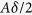 , where 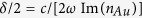 is the energy penetration depth. The absorptance of the ground plane is then given by:


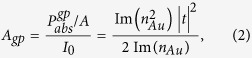


where *t* is the field transmission into the ground plane.

## Additional Information

**How to cite this article**: Azad, A. K. *et al.* Metasurface Broadband Solar Absorber. *Sci. Rep.* **6**, 20347; doi: 10.1038/srep20347 (2016).
